# Ancora su la Dentizione della Sapienza

**Published:** 1853-07

**Authors:** 


*Ancora su la Dentizione della Sapienza.* Del Dottor Napoleone Martelli. Milano. Giuseppe Chinsi, 1852.

This treatise is an examination of the extensive physiological and pathological relations of the last act of the function of dentition. These are rather glanced at than investigated, and the author refers to previous papers of his, in the Gazetta Medica Italiana Lombardia.

He cites several cases to exhibit the extensive sympathies of the outbreaking wisdom teeth. The first of these is quite remarkable. A young woman had that peculiar melancholy, called *pathema eroticum,* and was thought by her neighbors to be bewitched, so as to demand the intervention of the curate to exorcise the evil demon which was tormenting her. All these unpleasant symptoms, however, disappeared, at the author's simple announcement that the disease would not be cured till the patient had attained her full complement of teeth.

The secondary disturbance here was manifestly uterine, and it waked up all those anomalous nervous symptoms which are so apt to accompany uterine derangements. In his consideration of the subject, the doctor draws a very sensible parallel between the first and last dentition. As, in the first, the sympathetic irritation will spend its force upon the intestines and the brain, because these organs in infancy are in a high state of functional activity, so in the second, we may naturally expect the uterus to sympathize very strongly, because that organ is, at that time of life, in its full physiological development, and the entire sexual system is at its highest point of excitability.

Other cases are cited, in which the difficult eruption of the wisdom teeth, awakened a great variety of persistent irritations. In some instances these sympathetic disturbances were of a very grave character. Simulated phthisis, jaundice, asthma, chlorosis, melancholy, hemeralopia and pleurodynia are some of the disturbances which Dr. Martelli has seen to depend upon this difficulty in dentition. In other words, hysteria and hypochondriasis may be awakened by this as by other peripheral irritations of the nervous system.

This is a line of investigation which ought to be followed up, and the public will, no doubt, be glad to hear again from Dr. Martelli upon this subject, and to see his observations tested by others.

